# LncRNA HCP5 enhances the proliferation and migration of cervical cancer via miR-216a-5p/CDC42 axis

**DOI:** 10.7150/jca.64730

**Published:** 2022-03-21

**Authors:** Xiaomin Li, Bingxin Chen, Anni Huang, Ci Ren, Liming Wang, Tong Zhu, Jinfeng Xiong, Wencheng Ding, Hui Wang

**Affiliations:** 1 Department of Gynecologic Oncology Cancer Biology Research Center, Tongji Hospital, Tongji Medical College, Huazhong University of Science and Technology, Wuhan, China.; 2 Obstetrics and Gynecology Department, Women's Hospital School of Medicine Zhejiang University, Hangzhou, China.

**Keywords:** HCP5, miR-216a-5p, CDC42, cervical cancer, proliferation, migration

## Abstract

To investigate the important roles of the cancer-promoting long non-coding RNAs (lncRNAs) in cervical cancer, the up-regulated lncRNAs and prognostic analysis were identified through Lnc2Cancer and Lncar. LncRNA-regulated miRNA and miRNA-target mRNA were analyzed based on starBase v2.0 and miTarbase to predict the lncRNA-miRNA-mRNA ceRNA network. Based on the above findings, the abnormally expressed histocompatibility leukocyte antigen complex P5 (HCP5) was identified in 31 cervical cancer patients through RT-qPCR. The stable cell lines were constructed to explore the effect of HCP5 on the promotion of cervical cancer and the regulatory role on the expression of miR-216a-5p and CDC42. Cell Counting Kit-8 (CCK8) assay, cell clone formation, and transwell assay were used to examine proliferation and migration ability of cervical cancer cells. The results displayed that the overexpression of HCP5 promoted cervical cancer cell proliferation and migration *in vitro*, and the elevated HCP5 can also promote tumor growth *in vivo*. Besides, RT-qPCR and western blot assay revealed that elevated HCP5 suppressed miR-216a-5p expression and then up-regulated the expression of CDC42. In contrast, knocking down HCP5 resulted in increased expression of miR-216a-5p and then downregulated the expression of CDC42. Rescue experiments also demonstrated that miR-216a-5p could in part intercept in promotion impact caused by HCP5 on cervical cancer cells. Above all, HCP5, as an oncogene, can promote proliferation and migration ability of cervical cancer via the regulation of the miR-216a-5p/CDC42 axis.

## Introduction

Cervical cancer is known as one of the most common malignant tumors of the female reproductive system 1. Although the incidence of cervical cancer is gradually decreasing with the popularization of cervical cancer screening and vaccination, many women still die from cervical cancer each year. In 2020, about 600,000 women were diagnosed with cervical cancer, and about 340,000 died from cervical cancer worldwide 2. Therefore, the treatment of cervical cancer remains a daunting task. At present, cervical cancer therapy is still limited to surgery, radiotherapy, and chemotherapy. And because the early symptoms of cervical cancer are not obvious, most people are diagnosed at advanced stages. For recurrent and advanced cervical cancer, cisplatin combined with chemotherapy is the main treatment option 3. Still, the remission rate of chemotherapy is only between 20% and 36%, and the survival of patients is less than 1 year 4, 5. To make matters worse, radiotherapy hyposensitivity and chemotherapy resistance causes recurrence, metastasis, and even death, which greatly affects the therapeutic effect and limits the clinical application of concurrent chemoradiotherapy. Therefore, there is a great need for a deeper understanding of the mechanisms of cervical cancer development and to search for novel treatment molecular targets to ameliorate therapy for cervical cancer patients.

LncRNA is one kind of the non-coding RNAs with over 200 nucleotides (nt), which has many structural features of the mRNAs 6, 7. They had no protein-coding capacity and would be localized to the cytoplasm and nucleus 8. Research has revealed that 76% of the human genome would be transcribed to generate a lot of different lncRNAs 9, 10. With the development of the high-throughput sequencing technologies, more bio-functions of lncRNAs had been found, which worked as miRNA sponges and affected downstream genes 11-14. And the aberrant expression of lncRNAs can be found to have a close relationship with malignant tumors 15-18. Although many lncRNAs have been defined as key regulators in malignant tumors, the association between lncRNAs and cervical cancer remains largely unclear. Therefore, more efforts should be made to explore the bio-function of lncRNAs in cervical cancer.

HCP5 is one lncRNA that has been shown to be associated with several diseases, especially in cancer. It was dysregulated in many cancers, including hepatocellular carcinoma, glioma, and colorectal cancer 19-21. Recent research has found that HCP5 was upregulated in neuroblastoma tissues and promoted neuroblastoma cells proliferation 22. However, the expression and functional role of HCP5 in cervical cancer still need to be further explored.

In our study, data of Lnc2Cancer and Lncar has shown that HCP5 was highly expressed in cervical cancer tissues and correlated with poor prognosis, but the potential regulatory mechanism remained unclear. The following RT-qPCR and clinical characteristics analysis verified the potential carcinogenic role of HCP5 in cervical cancer. To further investigate the regulatory role of HCP5, we constructed HCP5 overexpression and knockdown cell models. We found that HCP5 can promote tumorigenicity through HCP5/miR-216a-5p/CDC42 axis.

## Materials and methods

### Screening for up-regulated lncRNA in cervical cancer

From the online database Lnc2Cancer (http://www.bio-bigdata.net/lnc2cancer/), lncRNA is differentially expressed in cervical cancer and normal cervix. The verification method was set as a high reliability experiment, including qPCR or Western blot. Among the 228 differentially expressed lncRNAs, 154 up-regulated lncRNAs were obtained.

### Prognostic analysis of up-regulated lncRNA

Lncar (https://Lncar.renlab.org/) is a comprehensive resource for lncRNAs from Cancer Arrays, containing the relationship between lncRNA and prognosis in cancer patients. Based on cervical cancer relapse-free survival analysis, we obtained the top 100 lncRNAs associated with poor prognosis in order of Hazard-ratios.

### Prediction of lncRNA-regulated miRNA and miRNA-target mRNA

LncRNA-regulated miRNAs were identified in starBase v2.0 (http://starbase.sysu.edu.cn/star-base2/index.php). The selection criteria were that the number of supporting experiences was more than one and the number of cancer types (pan-cancer) was more than 5. The miRNA target genes were verified by strong experimental evidence (reporter assay or western blot) in the miTarbase database (http://Mirtarbase.cuhk.edu.cn/php/index.php). The comprehensive prediction results were presented by the lncRNA-miRNA-mRNA ceRNA network.

### Human tissues

31 human fresh cervical cancer tissues and their adjacent normal cervical tissues were collected from histologically confirmed cervical cancer patients at Tongji Hospital of Wuhan in China. Tissue specimens were immersed in EP tubes containing RNAlater (Invitrogen) immediately after collection and stored at -80 °C. We collected the clinical characteristics (including age, tumor size, differentiation grade, TNM stage) of these 31 participants for further analysis. All patients included in this study had no history of other malignant tumors and signed written informed consent. The research project was approved by the Ethics Committee of Tongji Hospital, Huazhong University of Science and Technology.

### Cell lines and cultures

Human cervical cancer cell lines (SiHa and HeLa) and 293T cell lines were purchased from the American Type Culture Collection (ATCC). The two cell lines were cultured in medium DMEM (11965084, Gibco) supplemented with 10% fetal bovine serum (Kang Yuan Biology). Cells were cultured in a 37 °C humidified incubator containing 5% CO_2_.

### RNA extraction and RT-qPCR

According to the manufacturer's instructions, the total RNA of the human cervical cancer tissues and cell lines were extracted using the TRIzol reagent (Invitrogen). According to the manufacturer's protocol reverse transcription (RT) was performed with the HiScript®II Q RT SuperMix (Vazyme, Nanjing, China). According to the manufacturer's instructions, the real-time quantitative polymerase chain reaction (qPCR) was performed using the BIORAD CFX96 Touch Real-time PCR System and iTaq universal SYBR Green (Bio-Rad, USA). The expression levels of HCP5, miR-216a-5p, and CDC42 were calculated with the 2^-ΔΔCt^ method. U6 and GAPDH were used as the internal references for target genes, respectively. The expression levels of the target genes were normalized to the corresponding control. Primers for miR-216a-5p and U6 were purchased from GUANG ZHOU RIBO BIOTECHNOLOGY Co., Ltd. The other primers were shown as follows: HCP5 forward primers: 5ʹ-GCTGGACGATTCTCCTCACACT-3ʹ, reverse primers: 5ʹ-CTCCTCTCCAGGCACAGGTAAT-3ʹ; CDC42 forward primers: 5ʹ-GCTCTAGACCCTTAAGGGGAGGAG-3ʹ, reverse primers: 5ʹ-GCTCTAGAAAAAATCCCTATTAACAC-3ʹ.

### Preparation and transfection of lentivirus

The HCP5 overexpression and knockdown lentivirus were constructed by Shanghai Genechem Co., Ltd. For the HCP5 overexpression lentivirus (lv-HCP5), the HCP5 sequence was cloned into the Ubi-MCS-SV40-firefly-Luciferase-IRES-Puromycin lentivirus. Its control lentivirus was named lv-con. For the HCP5 knockdown lentivirus (lv-shHCP5), the siRNA sequence of HCP5 GATTCTCCTCACACTTACAAT was cloned into the hU6-MCS-Ubiquitin-firefly-Luciferase-IRES-puromycin lentivirus. The negative-control scrambled sequence, TTCTCCGAACGTGTCACGT, was cloned into the same lentivirus vector (lv-shcon). All constructed lentiviruses were transduced according to the manufacturer's instructions. Finally, the positive cells were screened with puromycin (2 μg/ml) for one week.

### Preparation and transfection of miR-216a-5p mimic and inhibitor

The mimic and inhibitor of miR-216a-5p and the negative control RNAs were purchased from OBiO Technology (Shanghai) Corp., Ltd. The miRNA transfections were performed using Lipofectamine 3000 (Invitrogen) according to the manufacturer's instructions. MiR-216a-5p mimics sequence (5ʹ-3ʹ): UAAUCUCAGCUGGCAACUGUGA; mimics NC sequence: UCACAACCUCCUAGAAAGAGUAGA; MiR-216a-5p inhibitor sequence (5ʹ-3ʹ): UCACAGUUGCCAGCUGAGAUUA; inhibitor NC sequence: UCUACUCUUUCUAGGAGGUUGUGA.

### Cell proliferation assay

Every 100 µl medium DMEM resuspension with 4×10^3^ cells was seeded in 96-well plates and cultured for 24 h, 48 h, 72 h in a 37 °C incubator with 5% CO_2_. Cell proliferation was detected using the Cell Counting Kit-8 (Dojindo Molecular Technologies) according to the manufacturer's manual at each time point.

### Colony formation assay

Every 1 ml medium DMEM resuspension with 1000 cells was seeded in 12-well culture plates and maintained in a 37 °C incubator with 5% CO_2_ for 2 weeks. Then the clones were fixed with 4% paraformaldehyde for 15 min and stained with 0.1% crystal violet for 20 min. The image of colony formation was scanned.

### Transwell assay

To explore whether HCP5 could influence the migration ability of cervical cancer cells, a Transwell assay was performed using a Transwell chamber with 8.0 μm pores (Corning, USA). Cells were cultured in the upper chamber with medium DMEM without FBS, and the lower chamber contained fresh medium DMEM with 20% FBS. Twenty-four hours later, cells on the upper chamber were removed, and cells from the lower surface were fixed with 4% paraformaldehyde, stained with 0.1% crystal violet. Photographs were taken using cellSens Dimension (version 1.8.1, Olympus).

### Western blot assay

Cultured cells were lysed for 30 min in ice-cold RIPA lysis buffer (Servicebio, Wuhan, China) containing protease inhibitors (Servicebio, Wuhan, China). Protein concentration was measured by the Coomassie brilliant blue G-250 (BioFroxx; neoFroxx GmbH) staining method. Sodium dodecyl sulfate polyacrylamide gel electrophoresis (SDS-PAGE) was used for protein electrophoresis. The protein was transferred to a polyvinylidene difluoride membrane. The PVDF membrane was blocked in 5% BSA for 1h and then incubated with diluted primary antibodies for 12 h at 4 °C. After washing with TBST three times, the PDVF membrane was incubated in diluted HRP Goat Anti-Rabbit IgG (1:5000, A21020, abbkine) or HRP Goat Anti-mouse IgG (1:5000, A21010, abbkine) at room temperature for 1h. The proteins were detected by the ECL system (Bio-Rad) and analyzed by Image Lab (6.0.1). All primary antibodies were shown as follows: GAPDH (1:2500, rabbit, YM1235, Immunoway), CDC42 (1:200, mouse, sc-8401, SANTA CRUZ BIOTECHNOLOGY).

### *In vivo* animal experiments

Twenty 4-week-old female nude mice were purchased from Gempharmatech Co., Ltd and were randomly divided into four experimental groups. They are kept at the Animal Research Centre of Tongji Medical College, Huazhong University of Science and Technology. We inoculated SiHa cells overexpressing or knock-down HCP5 (SiHa-lv-hcp5, SiHa-lv-shhcp5) into the right hindquarters of nude mice subcutaneously to observe tumorigenesis, and the control group was inoculated in the same way. All the nude mice were killed, and tumor tissue was collected after 30 days for comparative analysis. The length and width of the tumor were measured, and the volume is calculated using the formula as follows: [(length x width)^2^]/2. Tumor weight was also measured. Our animal experiment research was carried out strictly in accordance with the “Guidelines for the Welfare of Experimental Tumor Animals”. All experimental protocols were approved by the Animal Care and Use Committee of Huazhong University of Science and Technology.

### Immunohistochemistry

Tumor tissues harvested from nude mice were fixed in 4% formaldehyde and embedded in paraffin for immunohistochemistry assay. Paraffin-embedded tissue sections were first dewaxed, hydrated, and antigenically repaired. After being washed in PBS, the sections were incubated in 3% H_2_O_2_ for 10 min at room temperature to inhibit endogenous peroxidase. Next, sections were continued to be washed in PBS and blocked with goat serum for 30min, and then incubated with the primary antibody of Ki67 (1:200, ab16667, Abcam) overnight at 4 °C. On the second day, incubation of secondary antibody, DAB development, hematoxylin staining of nuclei, and dehydration were carried out according to the experimental procedures of the immunohistochemistry kit (ZSGB-BIO, Beijing, China). Finally, tissue sections were sealed with Neutral Balsam and photographed with cellSens Dimension for analysis.

### Statistical analysis

The data were expressed as the mean ± SD. According to the data types, the student's t-test and Fisher's exact test were conducted to examine the differences between the groups. All the experiments were performed three times in duplicate. The statistical analyses were performed with GraphPad Prism 8.0. Differences were considered statistically significant at P<0.05.

## Results

### HCP5 were up-regulated in cervical cancer

**Figure 1:**
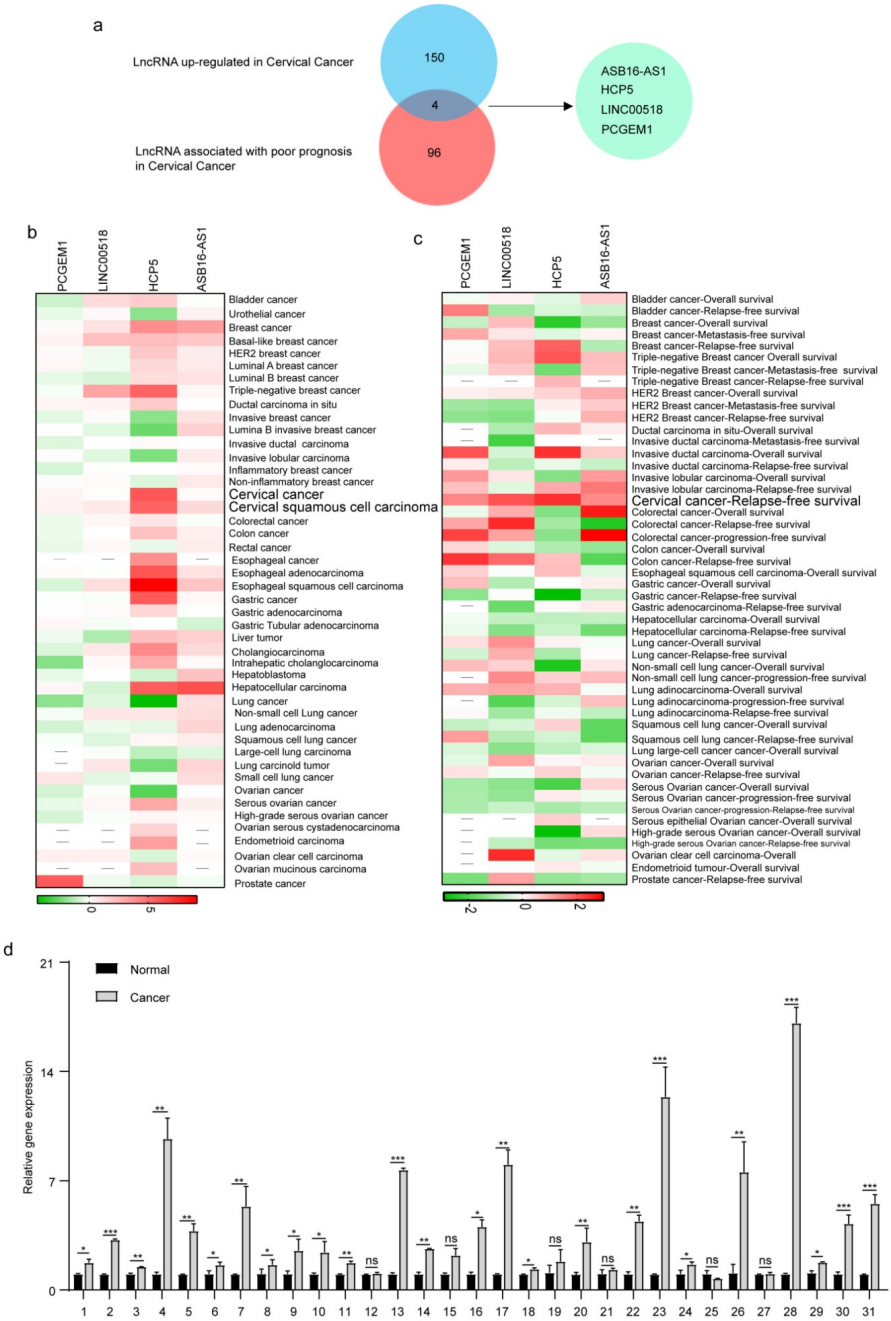
** HCP5 was upregulated in cervical cancer. (a)** Venn diagram of LncRNAs upregulated and LncRNA associated with poor prognosis in patients with cervical cancer. **(b, c)** The roles of 4 hub LncRNAs in 46 malignant tumors based on the lncar database. **(d)** The expression levels of HCP5 in the cancerous tissues and adjacent tissues from 31 cervical cancer specimens. ns, not significant; *P < 0.05, **P < 0.01, ***P<0.001.

To explore the lncRNAs which played an important role in cervical cancer patients, we used the Lnc2Cancer database to identify differentially expressed lncRNAs. After preliminary screening, we identified 228 dysregulated lncRNAs in cervical cancer, including 154 up-regulated lncRNAs. We further explored the lncRNAs related to cervical cancer patients' prognosis through Lncar, a comprehensive lncRNA database according to chip probes. After sorting by Hazard-ratios, we obtained the top 100 lncRNAs associated with poor relapse-free survival in cervical cancer patients. After crossing the up-regulated lncRNAs set with the poor-prognosis-related lncRNAs set, we got 4 hub lncRNAs, including ASB16-AS1, LINC00518, HCP5, and PCGEM1 (Figure 1a). Furthermore, we explored the roles of 4 hub lncRNAs in pan-cancer. We used Lncar to explore the different expression levels of 4 hub lncRNAs in 46 human cancerous tissues and corresponding paracancerous tissues. HCP5 was highly expressed in most malignant tumors, especially triple-negative breast cancer, cervical cancer, esophageal cancer, and hepatocellular carcinoma (Figure 1b). During exploring the effect of lncRNAs on survival, HCP5 was associated with a good prognosis in lung cancer, breast cancer, and ovarian cancer. Nevertheless, high expression of HCP5 led to poor prognosis in invasive ductal carcinoma and cervical cancer (Figure 1c). Furthermore, we used the 31 pairs of adjacent cancer and cancer tissues to test the expression levels of HCP5 using RT-qPCR. The results suggested that the expression levels of HCP5 in 25/31 cervical cancer tissues were higher than that of adjacent cancer (Figure 1d). The clinical characteristics of included patients were analyzed to evaluate the relationship between HCP5 and clinical characteristics in cervical cancer. We found that the expression level of HCP5 was significantly correlated with tumor size (P = 0.0041, Table 1). Taken together, these findings suggest that HCP5 may play a tumorigenic role in cervical cancer. Besides, we also detected that the expression of HCP5 was higher in SiHa and HeLa than that in 293T (Supplementary Figure 1). These results showed that HCP5 was up-regulated in cervical cancer.

### HCP5 enhanced the proliferation and migration of cervical cancer cells

**Figure 2:**
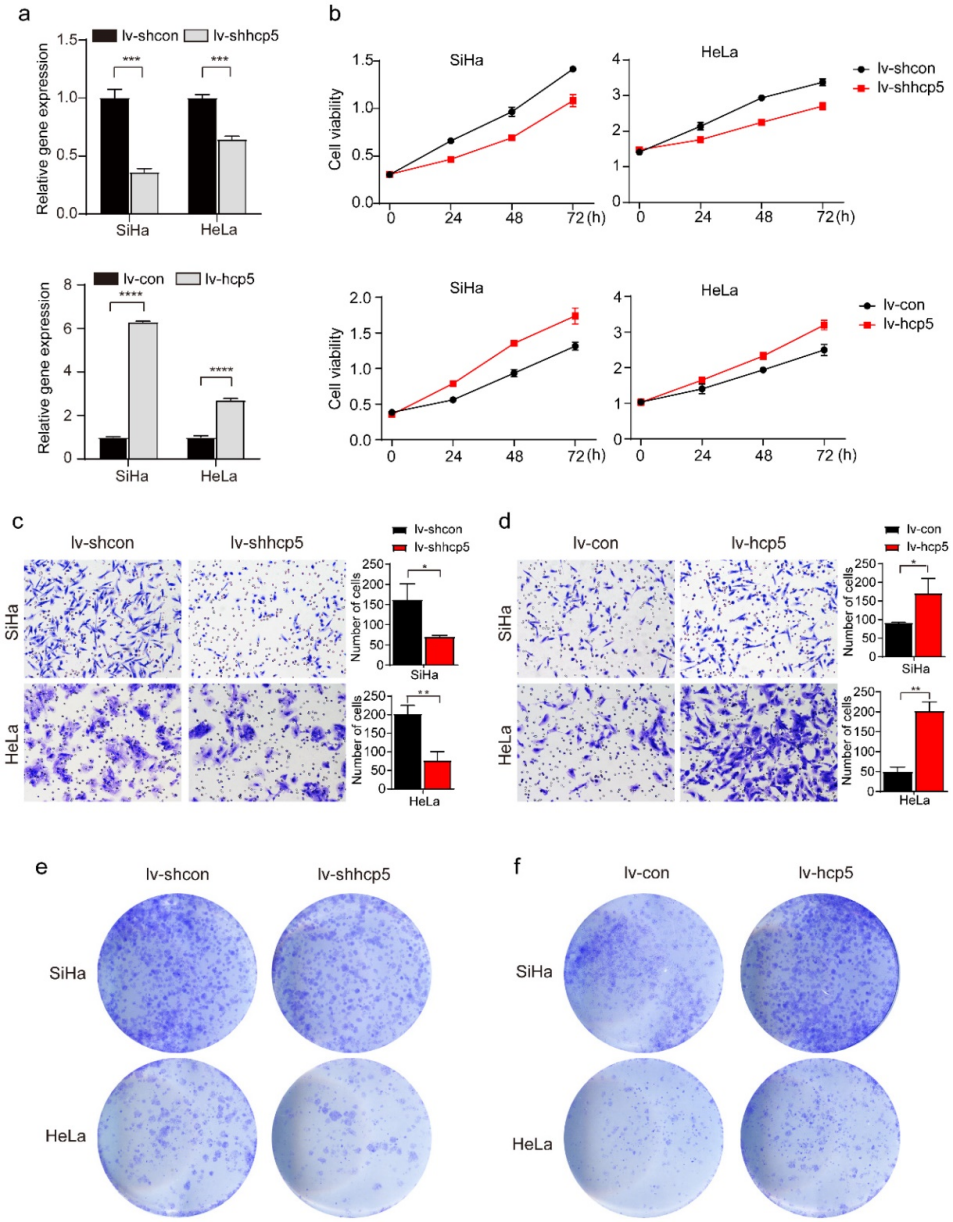
** HCP5 enhances the proliferation, migration, and clone formation of cervical cancer cells. (a)** The effect of HCP5 knockdown and overexpression in SiHa and HeLa cells by the transduction of the corresponding lentivirus. **(b)** CCK8 assay was used to assess the proliferative capacity of SiHa and HeLa cells at 24h, 48h, and 72h after knockdown or overexpression of HCP5. **(c, d)** Transwell assay was perfomed to detect the migration ability of SiHa and HeLa cells with knockdown or overexpression of HCP5 after being seeded in the upper chamber of Transwell for 24h. **(e, f)** Clone formation images of cells in SiHa and HeLa with knockdown or overexpression of HCP5. lv-shcon represents control of knockdown model of HCP5, lv-shhcp5 represents the knockdown model of HCP5, lv-con represents control of overexpression model of HCP5, lv-hcp5 represents overexpression model of HCP5. All the experiments were performed three times in duplicate. The data were presented as mean ± SD. **P<0.01, ***P<0.001, ****P<0.0001.

To further investigate the role of HPC5 in cervical cancer cells, HCP5 knockdown and overexpression models of SiHa and HeLa cells were constructed by the transduction of the corresponding lentivirus. The RT-qPCR results showed that stable HCP5 overexpression cervical cancer cells (lv-hcp5) and stable HCP5 knockdown cervical cancer cells (lv-shhcp5) were established (Figure 2a). Next, CCK8, Transwell, and clone formation assays were performed. The CCK8 experiment showed that overexpression of HCP5 promoted the proliferative ability of SiHa and HeLa cells. In contrast, these cells grew relatively slowly after HCP5 down-regulation (Figure 2b). Besides, Transwell assay also indicated that overexpression of HCP5 enhanced Siha and HeLa cells migration compared with the control cells (Figure 2c), while, knockdown of HCP5 inhibited the migratory ability of SiHa and HeLa cells relative to control cells (Figure 2d). Consistent with the results above, clone formation assay revealed that overexpression of HCP5 accelerated the clone formation of the cells (Figure 2e), while knockdown of HCP5 resulted in minimal clone formation (Figure 2f). These data indicated that HCP5 promoted cell proliferation and migration in cervical cancer cells.

### MiR-216a-5p and the target gene CDC42 were regulated by HCP5

**Figure 3:**
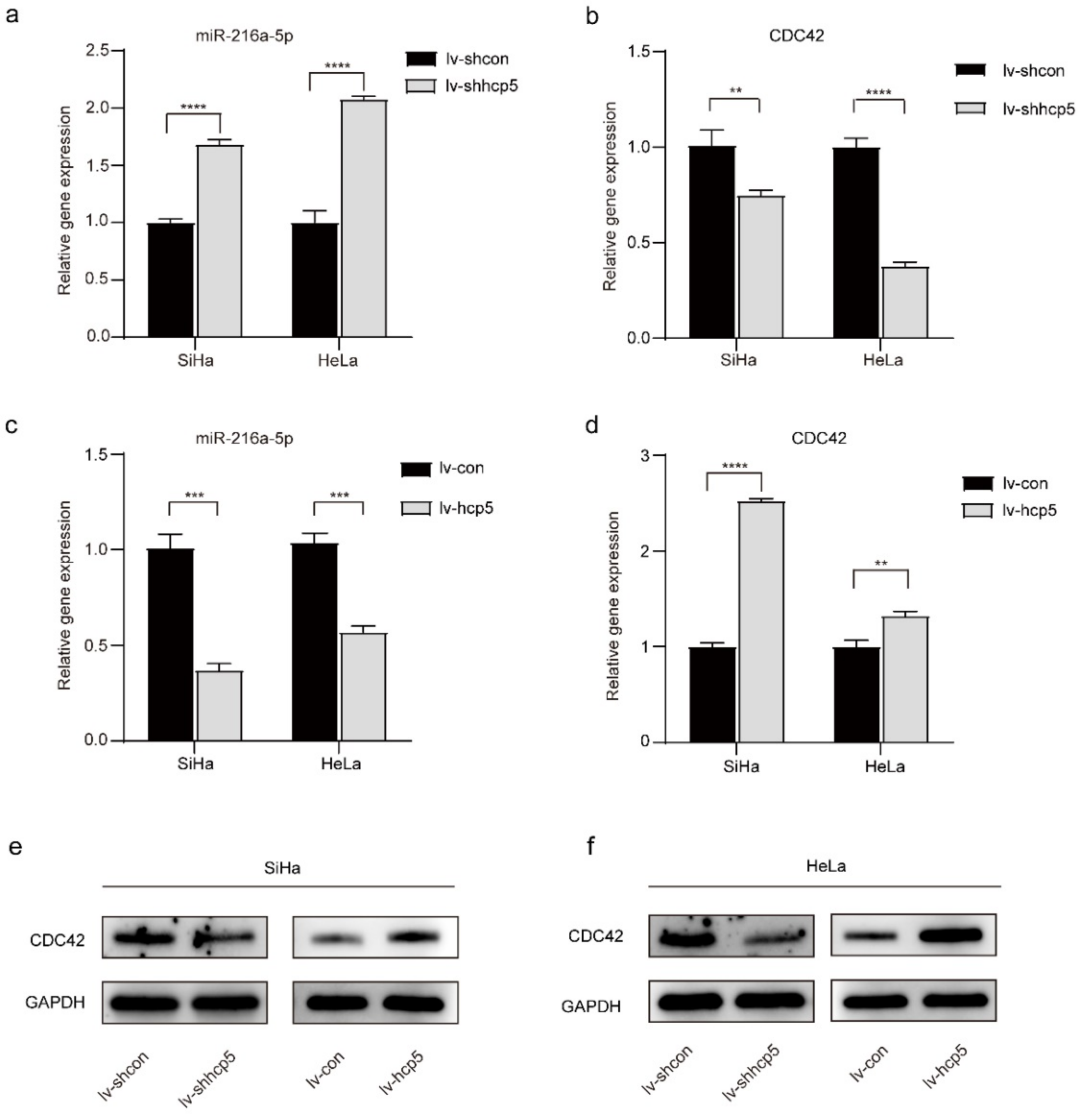
** Expression of miR-216a-5p and CDC42 is regulated by HCP5 in SiHa and HeLa cells. (a, b and c, d)** The RT-qPCR assay was used to analyze the relative gene expression of miR-216a-5p and CDC42 in SiHa and HeLa cells after overexpression or knockdown of HCP5. **(e, f)** Western Blot assay was performed to assess the expression of CDC42 protein in the established cells. All the experiments were performed three times in duplicate. The data were presented as mean ± SD. **P<0.01, ***P<0.001, ****P<0.0001.

LncRNAs are considered important regulators of the transcriptional and posttranscriptional processes of protein-encoding genes. Moreover, certain lncRNAs can regulate miRNAs associated with tumorigenesis. To further explore whether HCP5 affects the biological behavior of cervical cancer cells by regulating miRNAs, we queried starBase v.2.0, which predicted that miR-216a-5p interacted with HCP5 (Supplementary Table 1). Furthermore, target genes of each miRNA were predicted by reporter assay or western blot in the Mirtarbase database, which predicted that miR-216a-5p can regulate the expression of CDC42 (Supplementary Table 1, Supplementary Figure 1a). MiR-216a-5p has been investigated as a tumor suppressor in many cancers 23-26, and miR-216a-5p can negatively regulate the expression of CDC42 27, which was involved in cell growth and migration 28-30. Whether miR-216a-5p and CDC42 are involved in HCP5-induced growth and migration in cervical cancer was further investigated. The RT-qPCR experiment showed that the relative gene expression of miR-216a-5p was elevated after knockdown of HCP5 in SiHa and HeLa cells (Figure 3a), whereas the relative gene expression of CDC42 was down-regulated (Figure 3b). In contrast, when SiHa and HeLa cells overexpressed HCP5, miR-216a-5p expression was down-regulated (Figure 3c), and CDC42 expression was up-regulated (Figure 3d). Western Blot assay was also performed, the expression of CDC42 protein and mRNA expression levels are consistent (Figure 3e, f). The above experimental results illustrated that HCP5 negatively regulated miR-216a-5P and then positively regulated the expression of the target gene CDC42 in SiHa and HeLa cells.

### Up- or down-regulation of miR-216a-5p reversed CDC42 expression in cervical cancer cells with knockdown or overexpression of HCP5

**Figure 4:**
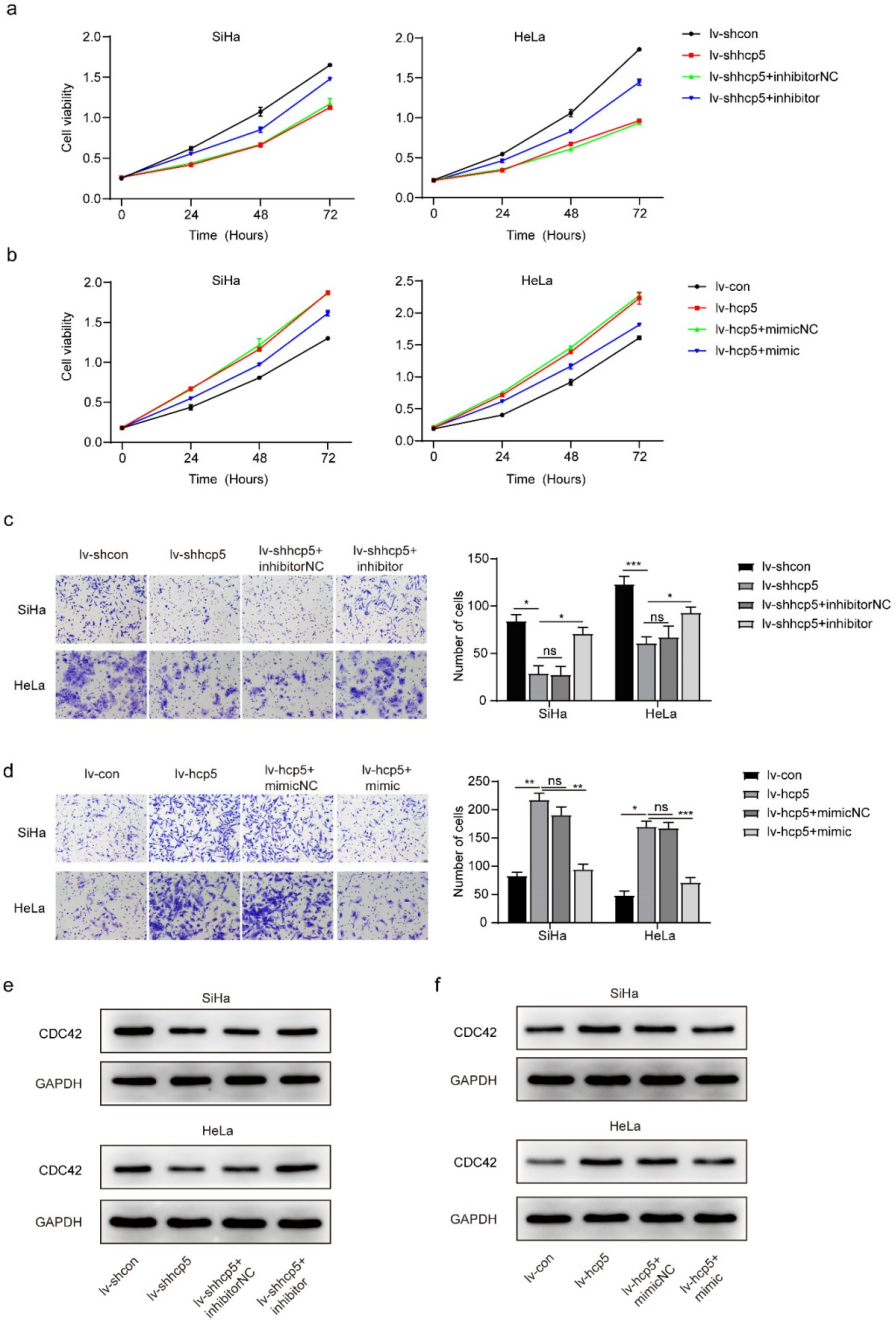
** The effect of HCP5 on proliferation and migration by targeting CDC42 in SiHa and HeLa cells was partially negatively regulated by miR-216a-5p.** CCK8 assay using the established cells treated with miR-216a-5p inhibitor (**a**) or mimic (**b**). **(c, d)** Cell migration was evaluated by transwell assay in different group cells. Western blot assay analyzed the protein expression of CDC42 in SiHa and HeLa cells with knockdown or overexpression of HCP5 after being treated with miR-216a-5p inhibitor (**e**) or mimic (**f**). All the experiments were performed three times in duplicate. The data was presented as mean ± SD, *P < 0.05, **P < 0.01, ***P<0.001.

To further illustrate that the expression of miR-216a-5p and CDC42 is regulated by HCP5 in SiHa and HeLa cells, we transfected the established cells with the miR-216a-5p mimic and inhibitor, respectively. Next, CCK8, transwell, and western blot assays were performed in different groups above. CCK8 assay showed that lv-shhcp5 cells with miR-216a-5p inhibitor grew faster than the lv-shhcp5 cells with miR-216a-5p inhibitor negative control (Figure 4a). While the lv-hcp5 cells with miR-216a-5p mimic grew more slowly than the lv-hcp5 cells with miR-216a-5p mimic negative control (Figure 4b). Data from transwell assay exhibited that down-regulation of miR-216a-5p could promote migration of lv-shhcp5 cells (Figure 4c), while up-regulation of miR-216a-5p inhibited migration of lv-hcp5 cells (Figure 4d). Besides, western blot results indicated that the expression of CDC42 protein was up-regulated in lv-shhcp5 cells treated with miR-216a-5p inhibitor compared with the miR-216a-5p inhibitor negative control (Figure 4e), the opposite phenomenon was found in lv-hcp5 cells treated with miR-216a-5p mimic compared with the miR-216a-5p mimic negative control (Figure 4f). The data above further revealed that HCP5 regulated the miR-216a-5p/CDC42 axis to enhance cervical cancer cell proliferation.

### HCP5 promoted cervical cancer tumor growth *in vivo*

**Figure 5:**
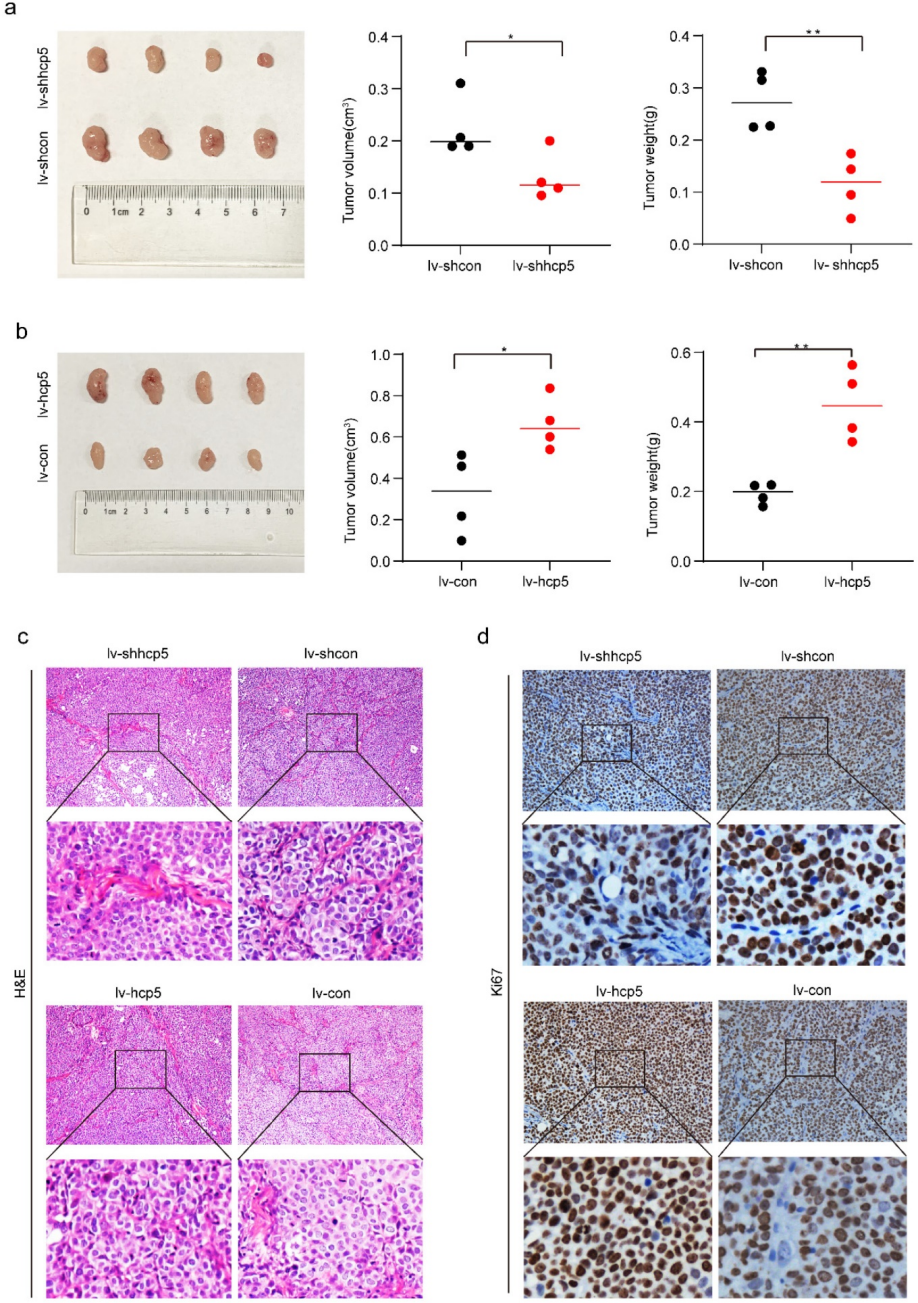
** HCP5 promoted tumorigenesis of SiHa cells *in vivo*. In the *in vivo* experimental analysis,** 5x10^6^ SiHa cells of different conditions were inoculated into the right posterior subcutaneous of nude mice. **(a, b)** Image of Xenograft tumors generated by SiHa cells and the corresponding tumor volume and tumor weight. **(c, d)** Representative images of IHC staining for H&E and immunohistochemical images showing the protein expression level of Ki67 in the tumor tissue samples. Four nude mice were included in each group of experiments. The data were presented as mean ± SD. *P<0.05, **P<0.01.

To further demonstrate the role of HCP5 in promoting tumorigenesis in cervical cancer, we inoculated SiHa cells of HCP5 overexpression or knockdown (SiHa-lv-hcp5, SiHa-lv-shhcp5) and their control group cells (SiHa-lv-con, SiHa-lv-shcon) into the right hindquarters of nude mice subcutaneously to observe tumorigenesis. Tumor tissue was collected after 30 days for comparative analysis. As shown in Figure 5a and Figure 5b, tumor volume in SiHa-lv-shhcp5 group was smaller than the control group. In contrast, tumor volume in SiHa-lv-hcp5 group was larger than the control group. And the results of comparative analysis of mean tumor weight in different groups were consistent with tumor volume changes. Besides, we also detected Ki67 expression in paraffin-embedded tumor tissues from nude mice tumors by immunohistochemical assay. The results showed that Ki67 expression was reduced in SiHa-lv-shhcp5 group tumors and elevated in SiHa-lv-hcp5 group tumors relative to the respective control group (Figure 5c, d). The above results suggested that HCP5 can promote the tumor growth of cervical cancer.

## Discussion

Cervical cancer seriously damages women's physical and mental health in our country. According to reports, more than 500,000 new cases are detected each year 31. Although researchers have made many efforts to identify cancer-promoting factors, the underlying molecular mechanisms of cervical cancer need further study. In recent years, lncRNAs have become a hot area of disease-promoting research, and a number of studies have identified the important roles of lncRNAs in cancer development 32, 33. Such as transcript antisense RNA (HOTAIR) has been found as an oncogenic lncRNA 34, which was associated with tumor growth, apoptosis, invasion, migration, and cell differentiation in many cancers 35-37. G-quadruplex-forming sequence containing lncRNA (GSEC) is also a prime example of oncogenic lncRNAs, that play an important role in the migration of colon cancer cells 38. As a cancer-associated lncRNA, HCP5 has been reported in numerous studies. It can produce a 2.5 kb transcript 39, 40 and is located between the MHC class I polypeptide related sequence A (MICA) and MHC class I polypeptide related sequence B (MICB) genes in the MHC I region of the chromosome 6p21.33 41. HCP5 is closely involved in some diseases, such as chronic kidney disease, HIV infection, autoimmune diseases 42-45. Moreover, an increasing number of studies have revealed the importance of HCP5 in tumorigenesis development, such as up-regulation of HCP5 could promote cell invasion and migration in human bladder cancer 46. Besides, dysregulation of HCP5 is also related to cell proliferation, apoptosis, and drug resistance in many other cancers 47-49. However, the role and molecular mechanism of HCP5 in cervical cancer progression remain poorly illuminated and need to be further explored. In our study, the results of databases and RT-qPCR have shown that HCP5 was upregulated in cancer tissues and correlated with poor prognosis, which indicates that HCP5 is related to cervical carcinogenesis. And further analysis of clinical characteristics suggested that the HCP5 level was significantly associated with tumor size. To further investigate the regulatory role of HCP5 in the progression of cervical cancer, we constructed HCP5 overexpression and knockdown cell models by lentiviral gene transfection vector for molecular mechanism study. Results showed that HCP5 enhanced the proliferation and migration of cervical cancer cells.

MicroRNAs (miRNAs) are small noncoding regulatory RNAs with approximately 17-25 nucleotides, which also participate in tumorigenesis 50, 51. MiR-34 has been regarded as a tumor-suppressive microRNA in prostate cancer by inhibiting EMT-associated migration and invasion 52, 53. And miR-1324 inhibits cell proliferation and invasiveness by targeting MECP2 in gastric cancer 54. Moreover, an increasing number of studies provide support for an extensive regulatory network inside ceRNAs, suggesting that ncRNAs shared miRNA binding sites could regulate target RNA by competing for posttranscriptional control 55, 56. We discovered that miR-216a-5p could directly interact with HCP5 through predictions of bioinformatics analysis and luciferase reporter assays from previous research 27. MiR-216a-5p has been investigated as a tumor suppressor in many cancers by targeting different mRNAs. For example, miR-216a-5p inhibits tumorigenesis by targeting TPT1 in pancreatic cancer 23 and targets TCTN1 to impede cell proliferation in esophageal squamous cell carcinoma 24. Our results revealed that miR-216a-5p was up-regulated in cervical cancer cells upon HCP5 knockdown, whereas overexpression of HCP5 resulted in a miR-216a-5p decrease. Rescue experiments also demonstrated that miR-216a-5p could in part intercept in promotion impact caused by HCP5 on cervical cancer cells. Our data verified that HCP5 could interact with miR-216a-5p to partly decrease its anticancer effect and promote cervical cancer progression.

MiRNAs posttranscriptionally regulate gene expression by recognizing complementary target sites of specific target mRNAs 51. Therefore, the downstream target mRNAs are an important part of the ceRNA regulatory network. We used Mirtarbase to predict the target of miR-216a-5p. CDC42 was chosen as our research focus and validated using western blot assay Based on the analysis above.

CDC42, a major member of the Rho GTPase family 57, has been shown in numerous studies associated with cell proliferation, cell cycle, invasion, and migration through different signaling pathways 58-60. For instance, a study found that CDC42 expression correlates positively with Ki-67 expression in breast cancer 61. Additionally, downregulation of CDC42 could exert crucial effects on migration and invasion of gastric cancer cells 62. It could also promote cell proliferation through PAK1/LIMK signaling pathway in cervical cancer 30. Hence, we hypothesized that HCP5 could compete with miR-216a-5p to upregulate CDC42 expression in cervical cancer. In this study, RT-qPCR and western blot demonstrated that lncRNA HCP5 promoted cervical cancer cell line proliferation and migration by inhibiting the expression of miR-216a-5p and resulting in the upregulation of CDC42.

With the development of high-throughput sequencing technology, cervical cancer's genomic and transcriptomic features have been fully characterized. Our study has found the key role of HCP5 in cervical cancer through the analysis of several databases, which is consistent with the previous study 63, 64. Subsequently, we found and verified the effect of the new regulatory mechanism of HCP5 on tumorigenicity of cervical cancer by *in vitro* and *in vivo* experiments. We also recognized the limitation of our study. Due to the relatively insufficient number of patients, only tumor size has a statistical difference in comparison between high and low expression groups when analyzing the relationship between clinical characteristics and HCP5. Therefore, in the follow-up study, we would expand the sample size to comprehensively study the impact of HCP5 on the clinical characteristics of cervical cancer patients.

In conclusion, our results illuminated that HCP5, an oncogenic lncRNA, is overexpressed in cervical cancer, and it can promote tumorigenicity of cervical cancer cells by upregulating CDC42 expression via miR-216a-5p. Therefore, HCP5/miR-216a-5p/CDC42 axis may serve as an essential promising therapeutic target for the clinical treatment of cervical carcinoma.

## Supplementary Material

Supplementary figures and table.

## Figures and Tables

**Table 1:** The clinical characteristics analysis in included 31 cervical cancer patients

Variable	Number	HCP5 expression		*P*-value
		High (25)	Low (6)	
**Age**				
≤ 40 years	12	9	3	0.6526
> 40 years	19	16	3	
**Tumor size**				
≤ 4 cm	14	8	6	0.0041**
> 4 cm	17	17	0	
**Differentiation grade**				
G1~G2	23	17	6	0.2976
G3	8	8	0	
**FIGO stage**				
I+II	23	18	5	>0.9999
III + IV	8	7	1	

**p<0.001.
