# Supplementary material for: LncRNA HCP5 enhances the proliferation and migration of cervical cancer via miR-216a-5p/CDC42 axis
Source: J Cancer. 2022 Mar 21;13(6):1882–94. doi: 10.7150/jca.64730 (PMC8990426; doi:10.7150/jca.64730)
Supplement: Supplementary file 1 — Supplementary figures and table. [file jcav13p1882s1.pdf]

Supplementary Information

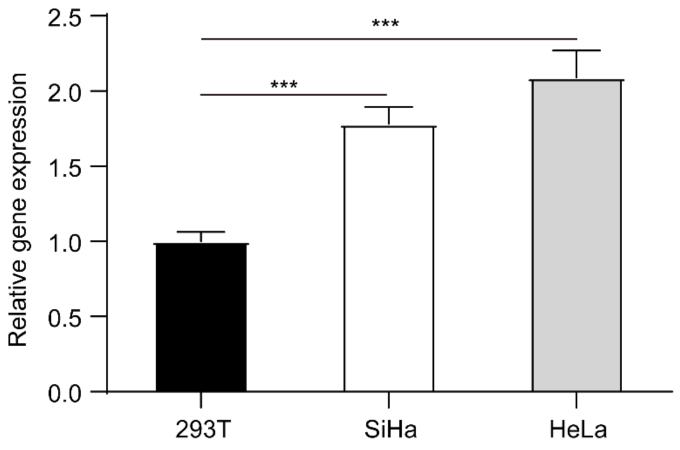

Supplementary Figure 1. The relative gene expression of HCP5 in 293T, SiHa and HeLa cells.

The experiment was performed three times in duplicate. The data was presented as mean ± SD, ***P＜0.001.

Supplementary Table 1.

lncRNA	miRNA	clipReadNum[1]	cancerNum[2]	targetgene[3]
HCP5	miR-17-5p	18	5	ZNFX1 CCL1 GPR137B NABP1 NPAT YES1 JAK1 PTEN CDKN1A PTPRO PKD2 BCL2L11 E2F1 MAP3K12 BCL2 MEF2D RUNX1 APP VEGFA MAPK9 DNAJC27 FBXO31 HIF1A TGFBR2 TNFSF12 BMPR2 CCND1 MYC NCOA3 SMAD4 ICAM1 SELE CCND2 E2F3 RB1 RBL1 RBL2 WEE1 RND3 SMURF1 TCF3 TCEAL1 HSPB2 MMP2 HBP1 SIRPA UBE2C TRIM8 DAPK3 PHLPP1 TP53INP1 ITGB8 BMP2 SOCS6 LIMK1 KAT2B TIMP3 ZBTB4 PDLIM7 STAT3 NPAS3 ZFYVE9 LDLR CLU ADAR MDM2 ETV1 EPAS1 TBC1D2 DNMT1 PKNOX1 TLR7 CLOCK ABCA1 VLDLR MFN2 EGR2 IGFBP3 TNF RAD21 PPP2R2A TP53COR1 BRCA2 CYP7B1
HCP5	miR-216a-5p	8	5	PTEN SIRT1 CDC42 CD44 SMAD7 BECN1 HNF4A CBL CEMIP JAK2 SDCBP KRAS CSNK2A1 HDAC8 PARP1 UGT2B7 PBK FOXM1 JUN

HCP5	miR-93-5p	18	6	TP53INP1 CDKN1A E2F1 MAPK9 VEGFA ITGB8 KAT2B TUSC2 PTEN PURA LATS2 RAB11FIP1 TGFBR2 STK11 CERS2 PHLPP2 SLC2A4 ATG16L1 DAB2 SMAD7 ZBTB4 CXCL8 RHOC ABCA1 RPS6KA4 PDCD4 ANG PTENP1 ZNRF3 FOXO3 MMP3 FOXA1
HCP5	miR-106b-5p	18	5	ITCH APP CDKN1A E2F1 KAT2B VEGFA BCL2L11 RB1 TCEAL1 CCND1 CCND2 E2F3 MAPK9 PTEN RBL1 RBL2 WEE1 EOMES PURA APC CASP7 JAK1 PKD2 SETD2 E2F5 ATG16L1 SMAD7 STAT3 ZBTB4 TWIST1 HIF1A TRIM8 CASP8 RUNX3 MMP2 MFN2 FAM129A RHOC PRRX1 FYN DAB2 SLC2A4 PTENP1 CYBB TNFRSF10A TNFSF11
HCP5	miR-27b-3p	18	8	NOTCH1 ST14 MMP13 ADORA2B CYP1B1 TRAPPC2B PPARG EDNRA EYA4 WEE1 VDR CYP3A4 PAX3 CCNT1 KHSRP PAX7 SEMA6A VEGFC CREB1 ABCA1 PSAP MFF LDLR FOXJ3 TGFBR1 CRISP2 DPYD SHC1 THBS2 THBS1 FOXO1 RET PHB CCNA2 NR2F2 RUNX1 SMAD2 HIP1R PLK2 NR5A2 ROR1 CCNG1 FZD7 OSBPL6 HMGB3 CDH11 EGFR MET CX3CL1 UCA1 PINK1 CDH5
HCP5	miR-106a-5p	18	5	E2F1 CDKN1A HIPK3 MYLIP RB1 APP RUNX1 ARID4B VEGFA IL10 FAS TGFBR2 CYP19A1 PTEN SIRPA SLC2A3 BMP2 STAT3 CCND1 ATM CASP7 BCL10 RUNX3 TIMP2 MAPK9 LIMK1 FASTK ULK1 HIF1A RBL2 APC MFN2 CXCL8 MYB ATG7 CDX2 MGST2 ERCC1 RND3 RARB

Supplementary Table 1. Prediction of HCP5-binding miRNAs using Starbase v.2.0 and target genes of predictive miRNAs using mirtarbase.1, ClipReadNum refers to the number of clipradeq reads. 2, CancerNum refers to the number of malignancies verified.

3, Validation methods of targetgenes are limited to strong evidence that only include reporter assay and western blot.

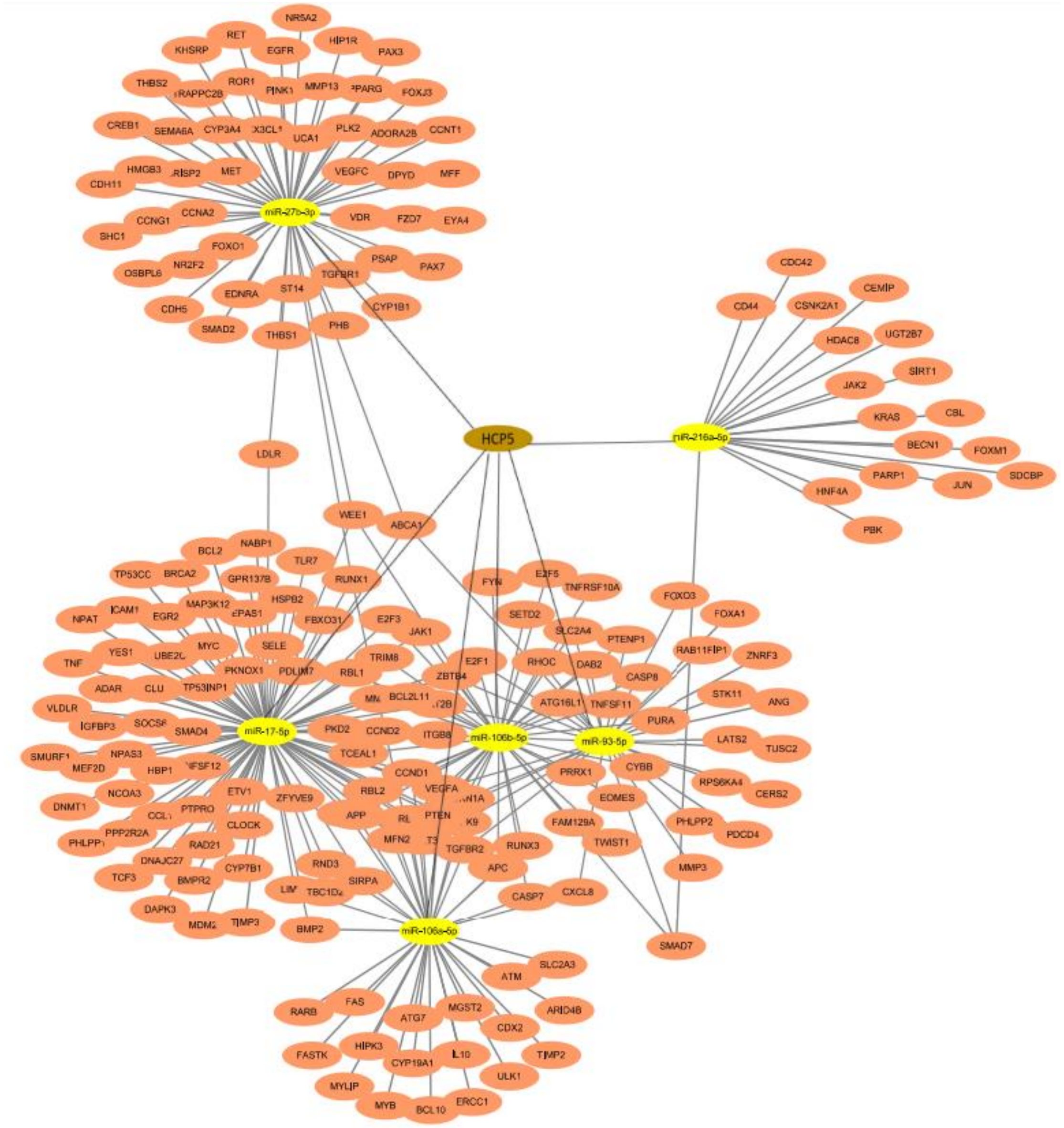

Supplementary Figure 2. The network of HCP5-binding miRNAs and relative target genes based on Starbase v.2.0 and mirtarbase.
